# Review of external parasites of small ruminants. A practical guide to their prevention and control by Peter Bates

**DOI:** 10.1186/1756-3305-5-93

**Published:** 2012-05-19

**Authors:** Olivier Sparagano

**Affiliations:** 1 School of Health, Community and Education Studies, Northumbria University, Newcastle upon Tyne, NE7 7XA, UK

## Review

It is always risky to write a book trying to review such a broad topic on ectoparasites bringing necessarily an update and some new information. Considering the number of international experts who apparently helped to share their knowledge I was hoping to read about the latest trials and epidemiological results.

However only around 5% of the references used in this book have been written in the last five years and that was extremely disappointing. Some pictures were taken more than 10 years ago, some being of poor quality or even blurry. All pictures are in black and white (except the one from the cover), which does not make this book very pedagogical and the pictures have no scale or geographical origin.

It is my general feeling after reading this book that the latest information is unfortunately not there. For ticks we learn about the Bm86 vaccine trials but nothing about what was done later with the Bm91/Bm95 and other concealed antigens. *Rhipicephalus microplus* is still called *Boophilus microplus* in this book and some trials presented were published more than 10 years ago. The diagnostic section is little better, with the ELISA section using references between 10 and 30 years old while molecular diagnostics are almost ignored.

Examples are presented depending on which international expert replied to the author and is very patchy. The latest acaricides and problems of resistance observed by many colleagues in the last few years are also not presented.

Considering the competition with published works elsewhere, and the resources found on the internet, this book does not provide enough value for money and does not cover recent trials, techniques and control products and would not answer the primary objectives of this book: to become a practical guide for prevention and control of such ectoparasites.

## Book details

**Bates P:** External Parasites of Small Ruminants. A practical guide to their prevention and control. CABI; 2012. 256 pages. ISBN 978 1 84593 664 8

## Competing interests

The author declares no competing interests.

